# Variations in cow milk and teat skin microbiota across the lactation cycle with intramammary cephalosporin use at dry-off

**DOI:** 10.1128/aem.02312-25

**Published:** 2026-05-05

**Authors:** Wannes Van Beeck, Mateus L. P. Lemos, Ashley M. Niesen, Peter Finnegan, Taylor M. Shih, Anhha Ho, Heidi A. Rossow, Maria L. Marco

**Affiliations:** 1 Department of Food Science and Technology, University of California 8789, Davis, California, USA; 2 Department of Bioscience Engineering, University of Antwerp 692679, Antwerp, Belgium; 3 Veterinary Medicine Teaching and Research Center, University of California 8789, Davis, California, USA; Universidad de los Andes, Bogotá, Colombia

**Keywords:** microbiome, skin, mastitis, *Staphylococcus*, antibiotics, milk

## Abstract

Cephalosporins and other broad-spectrum antibiotics are frequently administered prophylactically into the udder when dairy cows end their lactation cycle, termed dry-off, to reduce mastitis risk. However, the use of antibiotics on cows that do not have signs of infection may result in the selection of antibiotic-resistant microorganisms and negatively alter the udder microbiome. In this study, the effects of intramammary cephalosporin therapy with Cephapirin (CB) or Ceftiofur (CH) on milk and teat skin microbiota were examined for three dairies in California. Intramammary injections were given to healthy cows with high somatic cell counts (>200,000 cells/mL), indicative of infection. Samples were collected at dry-off (before treatment), 7 days later, and 55–75 days in milk (DIM) in the next lactation cycle. Dairy (milk: *R*^2^ = 6.22, skin: *R*^2^ = 7.56) and day of sampling (milk: *R*^2^ = 4.74 and skin: *R*^2^ = 3.77) had the highest impact on the milk and skin microbiota. CB or CH use was associated with a small but significant impact on milk microbiota beta-diversity (Bray-Curtis, *P* =0.003, *R*^2^ = 1.4%), but no effect was observed on the skin. At one dairy (Dairy 3), milk from cows receiving CB and CH had reduced proportions of *Staphylococcaceae* at 55–75 DIM compared to untreated cows. Overall, antibiotic use did not result in large significant (beneficial or harmful) changes in bacterial diversity in milk or on the teat skin; instead, the microbiota differences were mainly influenced by the time and location of sampling.

**IMPORTANCE:**

The use of antibiotics in agriculture is under increasing scrutiny due to the rising spread of antimicrobial-resistant bacteria. Our study showed that common preventative antibiotic intramammary treatment of cows with cephalosporins at the end of their lactation (dry-off) had minimal effects on the milk and teat skin microbiota of asymptomatic cows with high somatic cell counts.

## INTRODUCTION

Intramammary treatment with broad-spectrum antibiotics is frequently used as a preventive measure for dairy cows at the end of their lactation cycle (also called dry-off) to reduce the risk of infection and inflammation (1, 2). Up to 80% of dairies across the United States have adopted this prophylactic strategy (3). However, prophylactic, intramammary antibiotic use for cows that do not have signs of infection may pose a risk of increasing the spread of antimicrobial-resistant microorganisms and changes to the mammary microbiome, resulting in increased mastitis susceptibility in subsequent lactation cycles (4). Additionally, parenterally administered antibiotics have been detected in bovine milk up to 9 days after antibiotic treatment (5). Therefore, the necessity of routine antibiotic use at dry-off as a preventive measure has come under question.

Bovine milk from actively lactating cows contains diverse microorganisms belonging to multiple phyla, including the Bacillota (formerly known as Firmicutes), Pseudomonadota (Proteobacteria), Actinomycetota (Actinobacteria), and Bacteroidota (Bacteroides) (6). In Holstein cows, *Pseudomonas, Streptococcus, Acinetobacter,* and *Bacillus* were found to be frequently occurring genera in raw milk (7). The composition of milk microbiota changes during lactation, such as in notable increases in Actinomycetota late in lactation (8). In another study, proportions of *Staphylococcus* were higher late in lactation, whereas *Bacteroides* declined over time (9). The milk microbiota can also carry pathogens, such as with cows with (sub)clinical infections (10, 11). The cow teat skin harbors similar taxa as found in milk and includes species of *Streptococcus*, *Acinetobacter, Staphylococcus, Corynebacterium,* and *Turicibacter* (12). Teat skin microbiota may impact milk quality since microorganisms can be transferred into milk throughout lactation (12). Although the importance of commensal microorganisms in milk and on the teat skin is generally not known, previous studies have associated some genera, such as lactobacilli and *Paenibacillus,* with healthy cows and thus may contribute beneficially to the overall health of the cow (13, 14).

Subclinical bovine mastitis is one of the most common issues at dairy farms and is indicated by a decline in milk production, high somatic cell counts (SCC) (>200,000 cells/mL), but no clear sign of teat or udder inflammation (10, 11). High SCC results when there is an influx of somatic cells from the blood into the mammary gland (15) and suggests there is underlying inflammation and damage to milk-producing epithelial cells (16). Dairy cows are more prone to udder infection and high SCC during the dry-off period because of factors including slow mammary gland involution, late teat canal close-up, and increased milk retention (17–20). Cows with subclinical mastitis may eventually progress to clinical mastitis or become chronic carriers if left untreated (21). Subclinical mastitis is often accompanied by the presence of several opportunistic pathogens, such as non*-aureus* staphylococci, *Escherichia coli,* streptococci, and *Corynebacterium* spp (22–24). Because of these risks, cows in the United States frequently receive intramammary injections of either Cephapirin (CB), a first-generation cephalosporin (25), or Ceftiofur (CH), a third-generation cephalosporin (26), at dry-off irrespective of SCC status. Both cephalosporins are characterized by their broad activity against both Gram-positive bacteria (such as *Staphylococcus*) and Gram-negative bacteria (such as *E. coli*, *Klebsiella* spp., and *Enterobacter* spp.) (27). Thus, while cephalosporins can inactivate mastitis-causing pathogens, the antibiotics may also harm beneficial commensal microbes in milk or on the teat skin, resulting in a drug-induced microbiota perturbations.

**Fig 1:**
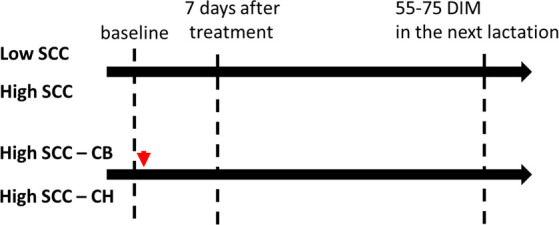
Study design. Milk and teat skin swab samples were collected from three dairies in California, encompassing three timepoints (baseline [dry-off], 7 days later, and 55–75 DIM in the next lactation cycle) and four treatment groups (low and high SCC) controls and high SCC cows given either CB or CH after baseline collection. The red arrow indicates antibiotic treatment.

Herein, we characterized the microbiota in freshly expelled bovine milk and on the teat skin from which the milk was obtained at dry-off (baseline), 7 days later, and after 55–75 days in milk (DIM) in the next lactation (Fig. 1) at three dairies in California. At each dairy, groups of cows (10 per group) with high, but subclinical levels of SCC (>200,000 cells/mL) at baseline received an intramammary prophylactic administration of CB or CH or were not treated with antibiotics (high SCC controls). Cows with low SCC (<200,000 cells/mL; 10 per group, low SCC controls) were included for comparison. The strength of the study lies in its multifactorial approach, including the sampling of milk and skin microbiota from cows at different dairies over time to detect long-term, potentially detrimental shifts in microbail composition in response to prophylactic antibiotic (CB or CH) treatment.

## MATERIALS AND METHODS

### Experimental design

A total of 372 milk and teat skin swab samples were collected for analysis from three dairies in California, encompassing three timepoints (baseline [dry-off], 7 days later, and 55–75 DIM in the next lactation) and four treatment groups (low and high SCC controls and high SCC cows given either CB or CH) (Fig. 1). Cows were enrolled in the study if they were greater than 210 days pregnant or if they produced <20 kg of milk per day. At baseline, milk samples were obtained from each quarter and SCC quantified by the Tulare County Dairy Herd Improvement Association using a Fourier Transform Spectrometer 600 Combi System (Combi; Bentley Instruments, Chaska, MN, USA). Cows were placed in the low SCC group if the SCC was <100,000 cells/mL in all four quarters. If SCC were >200,000 cells/mL in at least one-quarter, cows were placed in the high SCC group.

Once cows were grouped by SCC, they were assigned to one of four treatments (*n* = 10 cows per group): low SCC Controls; high SCC Controls; high SCC with Cephapirin Benzathine (CB) (300 mg ToMorrow; Boehringer Ingelheim Vetmedica Inc., St. Joseph, MO); and high SCC with Ceftiofur Hydrochloride (CH) (500 mg Spectramast DC; Zoetis Inc., Kalamazoo, MI). All treatments were administered by dairy employees. After baseline sample collection and prior to infusion of the cephalosporins, the teats were dipped with an antiseptic (pre-dip), dried with a clean towel, and milk was expelled. The CB and CH treatments were then provided by gently inserting a nozzle containing CB or CH into the teat canal. Cephalosporin was infused (300 mg CB and 500 mg CH), and the teat was briefly massaged. Control treatments received no cephalosporin antibiotics or placebo. Notably, although at least 10 cows were enrolled in each group, 18 cows were removed over the course of the study because of low milk yields, or they were no longer available (sold due to low milk yield, died, or had an abortion) (Fig. S1).

### Teat skin swab and milk collection

The quarter (teat) with the highest SCC per cow was selected for milk and teat swab sample collection and analysis. Cow teats were swabbed with a sterile 2 × 2 gauze moistened with sterile NaCl (0.9% wt/vol)-Tween 80 (1% vol/vol) solution. Sterile hemostats were used to remove the gauze from the tube, swab the teats, and then place the swab into a 50 mL tube for storage. For milk collection, the external teat surface was first wiped with gauze soaked in 70% ethanol. A total of 50 mL milk was collected by a gloved hand into separate tubes; 25 mL for microbiota analysis and 25 mL for compositional dairy herd improvement (DHI) analysis. For the latter, milk composition (percent milk fat, milk protein, lactose, solids not fat, and milk urea nitrogen and SCC [K/mL]) was measured by the Tulare County Dairy Herd Improvement Association using a Fourier Transform Spectrometer 600 Combi System (Combi; Bentley Instruments, Chaska, MN, USA).

Swabs and milk were transported overnight on ice to the Marco laboratory at UC Davis. Upon receipt, the swabs were stored at −20°C until dislodging the bacteria by incubation in cold phosphate-buffered saline (PBS, pH = 7.2; 137 mM NaCl, 2.68 mM KCl, 10.1 mM Na_2_HPO_4_, 1.76 mM KH_2_PO_4_) on ice with intermittent 10 s of vortexing for 3 min. Cells were collected by centrifugation at 13,000 × *g* for 10 min at 4°C and washed twice in PBS before the resulting cell pellets were frozen at −20°C. For the milk, immediately after receipt at UC Davis, the milk was centrifuged at 13,000 × *g* for 5 min at 4°C. Cell pellets were then washed twice in PBS before storing at −20°C.

### DNA extraction

#### Teat swabs

Frozen cell pellets were suspended in lysis/binding solution concentrate included in the MagMax Total Nucleic Acid Extraction Kit (Thermo Fisher Scientific, Waltham, USA), homogenized with 0.1  mm zirconium beads (Thermo Fisher Scientific, Waltham, USA) using a Fast-Prep−24 Instrument (MP Biomedicals) for 10 s at 4 m/s, and then centrifuged for 10 min at 10,000 × *g*. This lysis procedure was previously shown to be effective at retaining an accurate distribution of bacterial taxa in a mock community (28). DNA was then purified from the supernatant using the MagMax Total Nucleic Acid Extraction Kit following the manufacturer’s protocol. DNA was stored at −20°C.

#### Milk

The protocol used for DNA extraction from swab samples did not result in amplifiable DNA from the freshly expelled milk (data not shown). Therefore, a cetyltrimethylammonium bromide (CTAB) DNA extraction protocol, modified after (29), was used. In short, cell pellets were suspended in 1.2 mL CTAB extraction buffer (100 mM Tris pH 8.0, 1.4 M NaCl, 20 mM EDTA pH 8.0, 5% wt/vol polyvinylpyrrolidone, 2% wt/vol CTAB, and 1% vol/vol beta-mercaptoethanol). The suspension was homogenized in 0.1  mm zirconium beads in a Fast-Prep−24 Instrument for 45 s at 6 m/s, and the resulting suspension was centrifuged for 10 min at 10,000 × *g*. The supernatant was collected and incubated at 65°C for 30 min, with tube inversions every 10 min, prior to centrifugation at 13,500 × *g* for 10 min. Phenol:chloroform:isoamyl alcohol (24:24:1, Thermo Fisher Scientific, Waltham, USA) was added to the lysate and incubated at room temperature for 15 min. This step was repeated, and then the upper phase was collected after centrifugation at 13,500 × *g* for 10 min for purification with the PowerSoil DNA Isolation Kit (MoBio, Carlsbad, CA, USA) according to the manufacturer’s instructions.

### 16S rRNA gene amplicon DNA sequencing and analysis

PCR was carried out using Extaq polymerase (TaKaRa Bio Inc, San Jose, USA) and the 16S rRNA gene V4 hypervariable region (30) 515F (5′-XXXXXXXXCACGGTCGKCGGCGCCATT-3′) and 806R (5′-GGACTACHVGGGTWTCTAAT-3′) primers, including barcodes (random 8-bp barcode) for multiplexing samples attached to the 5′ end of the forward (515F) primer (31, 32). PCR was performed under the following conditions: denaturation at 94°C for 3 min, 30 cycles of 94°C for 45 s, annealing at 55°C for 60 s, and elongation at 72°C for 30 s. PCR amplicons were pooled and eluted from a 1% agarose gel using the Wizard SV Gel and PCR Clean-Up Kit (Promega, Madison, USA). The Ion Plus Fragment Library Kit (Thermo Fisher Scientific, Waltham, USA) was used for library preparation, and library quality was assessed using a 2100 Bioanalyzer (Agilent, Santa Clara, USA). On-chip library preparation was performed with the Ion Chef Instrument (Thermo Fisher Scientific, Waltham, USA). An Ion Torrent S5 (Thermo Fisher Scientific, Waltham, USA) was used for DNA sequencing in three different runs, ensuring that samples from different dairies were present on each run to avoid batch effects. A total of 5,709,613 and 12,760,376 reads were collected for milk and teat skin 16S rRNA V4 amplicons, respectively. A total of 37 milk and 64 skin samples were excluded because they did not yield sufficient reads for further processing (Fig. S1).

DNA sequences obtained were processed separately from each sequencing run. Demultiplexed, single-end reads were imported into QIIME2 (33). The first 21 bases at the 3′ end were trimmed, and reads were truncated at 290 bp. Amplicon sequence variants (ASVs) were identified through denoising using DADA2 (34). A feature classifier based on the SILVA v.138 V3-V4 regions database (35) using the Naive Bayes method was applied for taxonomic classification. Downstream analyses were carried out in R v. 4.1.3 (36) using Tidytacos (37) for visualizations. Inverse Simpson and Observed indexes were calculated for alpha-diversity, whereas the Bray-Curtis metric was used to calculate the distance matrices and plot the Principal Coordinate Analysis (PCoA) for beta-diversity.

### Statistical analysis

Due to the sparsity and non-normality of the sample distribution, the non-parametric Wilcoxon test was used for pairwise comparisons between treatments, including the Benjamini-Hochberg multiple testing correction. For beta-diversity analysis, permutational multivariate analysis of variance (PERMANOVA) was used to test the impact of environmental factors and metadata on the milk and skin microbiota. This test was performed using the adonis function within the vegan package, version 2.6.10 (38). Results were deemed significant if an adjusted *P*-value <0.05 was obtained.

## RESULTS

### Milk and teat skin have distinct microbiota

**Fig 2:**
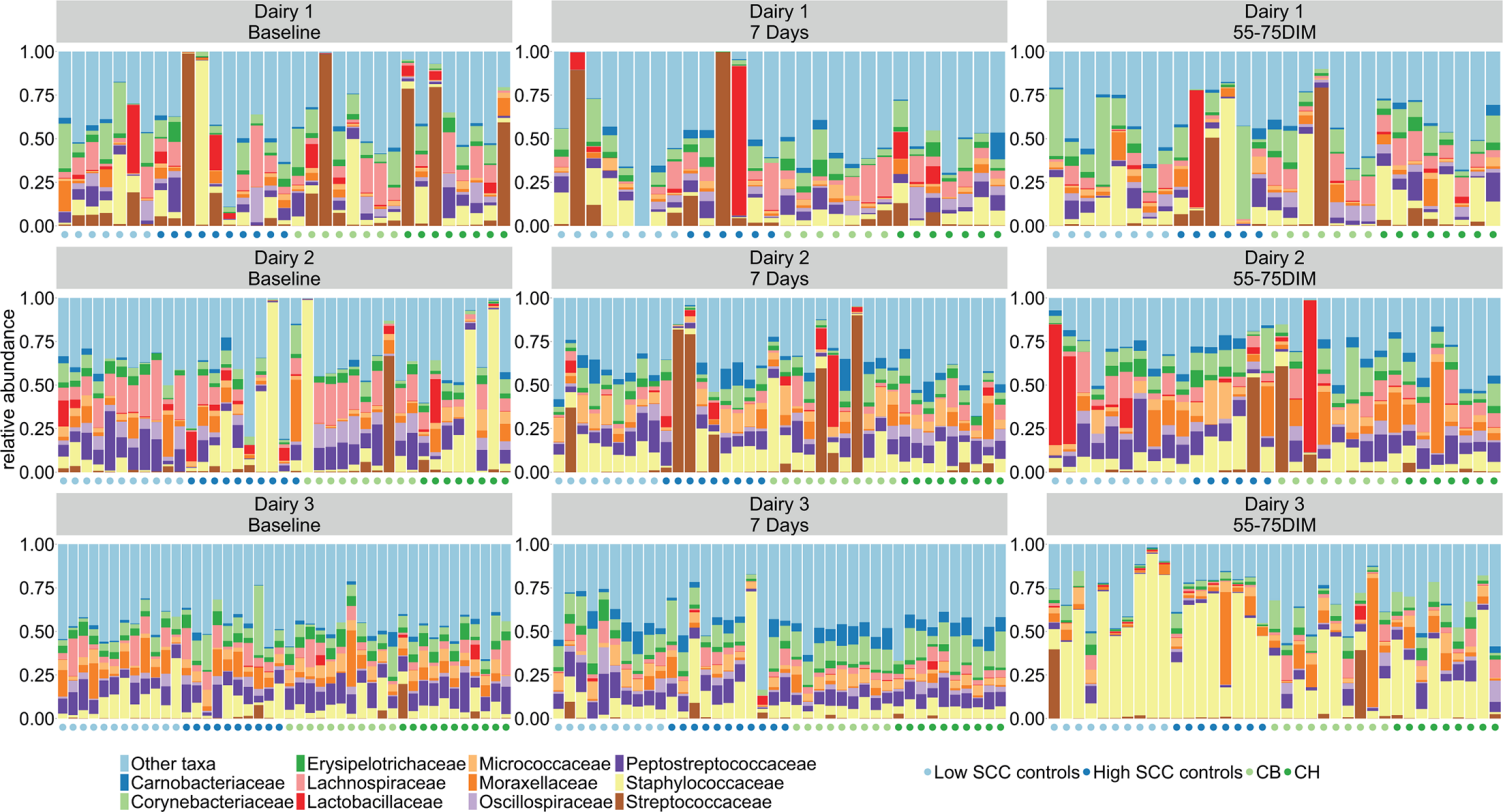
Bacterial taxa in freshly expressed milk across dairies, treatments, and sampling timepoints. The 11 most abundant families are shown. Samples are clustered based on treatment group, as indicated below each bar. Missing or low-quality read samples are not shown.

Freshly expelled milk and teat skin swabs sampled at baseline (at dry-off and before antibiotic use) were compared to determine whether the milk and teat skin microbiota were sufficiently similar that the impacts of antibiotic use could be measured for the two sample types combined. Freshly expelled milk collected at baseline was diverse, with an average ± standard deviation of 64 ± 25 families (102 ± 48 genera) across the samples. Approximately one-third (24 families) comprised an average relative abundance above 1% per sample. Despite this significant intrasample microbial diversity, a core milk microbiota (defined as a 90% prevalence and an average relative abundance of >1%) consisting of 11 bacterial families was found (Table 1). These families comprised an average relative abundance of 54.93% in individual milk samples at baseline. *Staphylococcaceae* (11.70% ± 18.5%; average ± standard deviation across all samples), *Peptostreptococcaceae* (8.70% ± 6.23%), and *Lachnospiraceae* (8.41% ± 7.96%) had the highest mean relative abundance (Fig. 2).

**TABLE 1:** Core microbiota^*a*^ in milk and on the teat skin at dry-off

Bacterial family	Prevalence^*b*^	Mean relative abundance (%)^*c*^
Milk samples		
*Staphylococcaceae*	93.58	11.24
*Peptostreptococcaceae*	100.00	8.85
*Lachnospiraceae*	96.33	8.60
*Corynebacteriaceae*	97.25	6.35
*Moraxellaceae*	99.08	4.74
*Erysipelotrichaceae*	96.33	3.54
*Oscillospiraceae*	95.41	3.49
*Micrococcaceae*	94.50	3.33
*Clostridiaceae*	91.74	2.18
*Ruminococcaceae*	91.74	1.42
*Bifidobacteriaceae*	91.74	1.19
TOTAL		54.93
Skin samples		
*Moraxellaceae*	97.89	14.59
*Corynebacteriaceae*	100.00	14.18
*Staphylococcaceae*	96.84	7.43
*Peptostreptococcaceae*	93.68	6.12
*Lachnospiraceae*	100.00	5.07
*Micrococcaceae*	100.00	3.35
*Erysipelotrichaceae*	90.53	2.98
*Oscillospiraceae*	92.63	2.75
*Bacillaceae*	90.53	2.53
*Aerococcaceae*	94.74	2.52
*Bacteroidaceae*	93.68	1.26
*Clostridiaceae*	94.74	1.23
*Streptococcaceae*	94.74	1.17
*Dietziaceae*	98.95	1.11
TOTAL		66.29

^*a*^ To be included in the core microbiota, the family was required to be present in at least 90% of the milk or teat swab samples at all three dairies and at a relative abundance above 1% at baseline (dry-off).

^*b*^ Prevalence of the bacterial families across all milk and skin samples at baseline.

^*c*^ Mean relative abundance of bacterial families per milk and skin sample at baseline.

**Fig 3:**
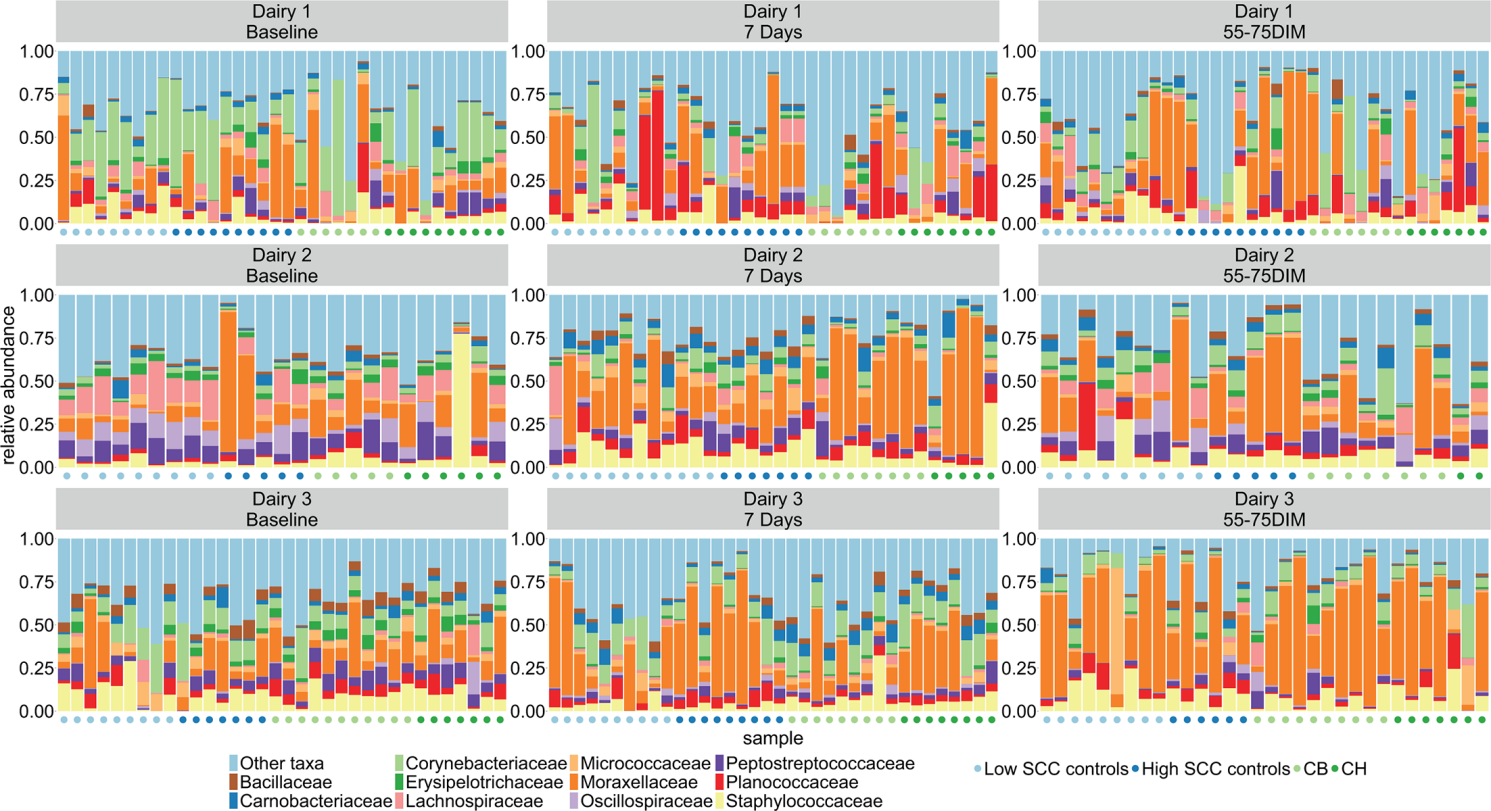
Bacterial composition on teat skin across dairies, treatments, and sampling timepoints. The 11 most abundant families are shown in a stacked bar graph. Samples are clustered based on treatment group, as indicated below each bar. Missing or low-quality read samples are not shown.

The teat skin had an even more diverse microbiota than milk at baseline, with 84 ± 26 families (49 ± 55 genera; average ± standard deviation across all samples) recovered from each skin sample. Approximately one-fifth (19 families) were present in proportions greater than 1%. The teat skin core microbiota consisted of 14 families, comprising an average total proportion of 66.29% per sample (Table 1). On the skin, *Moraxellaceae* (14.59% ± 16.26%; average ± standard deviation across all samples)*, Corynebacteriaceae* (14.18% ± 13.73%), and *Staphylococcaceae* (7.43% ± 7.45%) had the highest mean relative abundance (Fig. 3).

**Fig 4:**
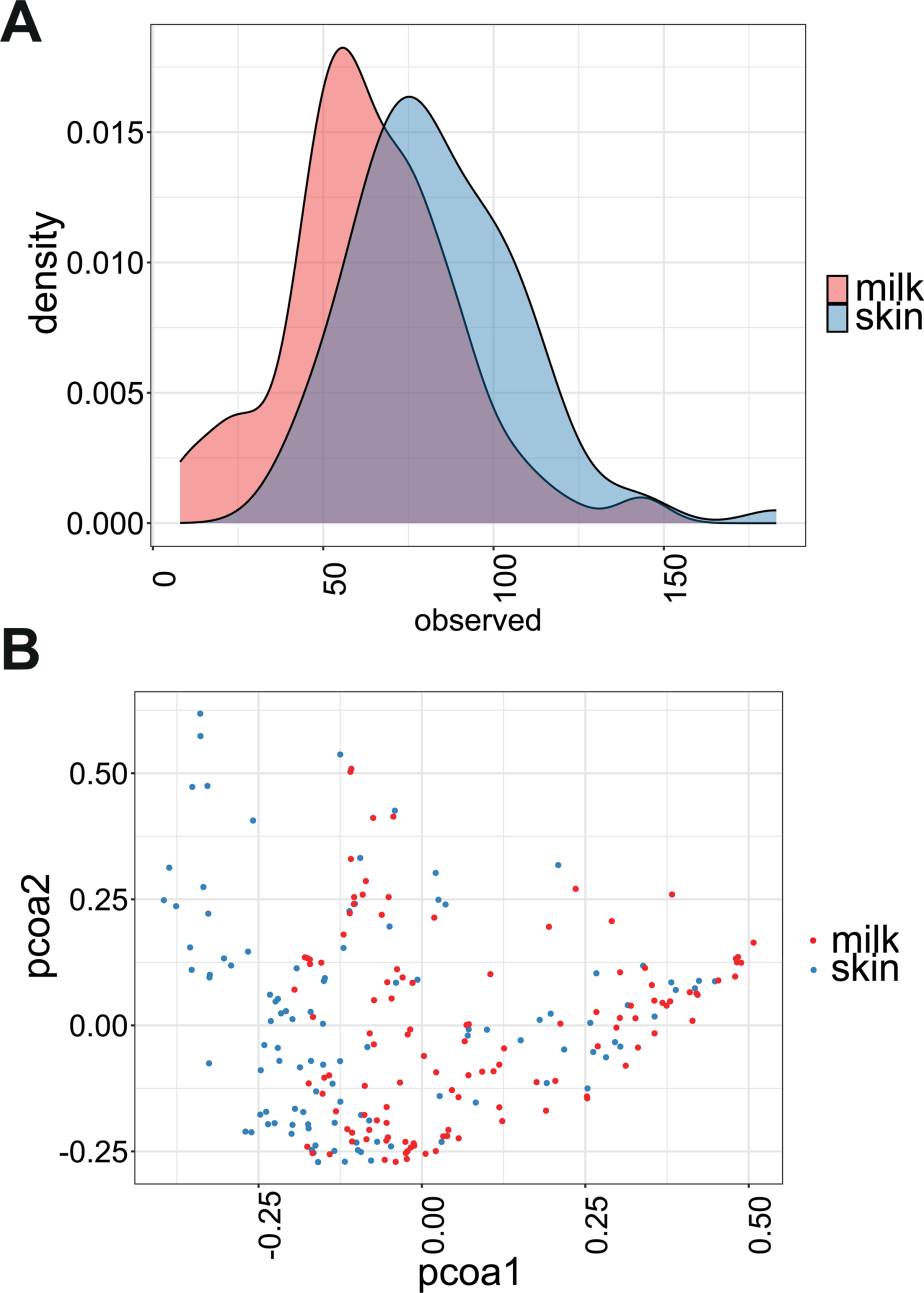
Milk and teat skin harbored a diverse but distinct microbiota at dry-off. (**A**) Density plot of the observed alpha-diversity of bacterial families in milk and on teat skin at baseline (dry-off). Alpha diversity was calculated as the number of observed families in each milk or teat skin sample at baseline. (**B**) PCoA was performed using the Bray-Curtis dissimilarity matrix on ASVs at the family level.

Comparisons between the microbiota in milk and on the skin at baseline showed that milk had a lower bacterial alpha-diversity compared to skin (Fig. 4A). Variations between the microbiota at the two sampling sites were confirmed by assessment of bacterial beta-diversity, revealing a clear distinction between the milk and skin microbiota (Bray-Curtis; ANOSIM, PERMANOVA, *P*<0.05, Fig. 4B). These differences were consistent across the three dairies and timepoints (Fig. S2). At the taxonomic level, nine of the core microbiota families were shared between the milk (9 out of 11) and teat skin (9 out of 14). However, milk contained significantly higher proportions of *Clostridiaceae, Lactobacillaceae, Lachnospiraceae,* and *Oscillospiraceae,* and skin had higher proportions of *Corynebacteriaceae* and *Moraxellaceae* (*P* < 0.05, Fig. S3). At the genus level, milk had significantly higher proportions of lactobacilli and *Staphylococcus* and lower proportions of *Corynebacterium* and *Acinetobacter* compared to the teat skin (*P* < 0.05, Wilcoxon, Fig. S4). Thus, according to the methods applied here, despite having some overlap, the bacteria in freshly expelled milk were distinct from those on the surrounding teat skin.

### Correlation between the milk and skin microbiota and milk quality at baseline

We next assessed whether the milk and teat skin microbiota were correlated with milk composition parameters measured at baseline (dry-off). Small significant correlations were found between SCC levels and bacterial beta-diversity in milk (PERMANOVA, *R*^2^= 3.3% *P* = 0.003), but not with the bacterial beta-diversity on teat skin (PERMANOVA, *R*^2^ = 0.6%, *P* = 0.7). Similarly, SCC was negatively correlated with the inverse Simpson alpha-diversity of the bacteria in milk (Pearson’s correlation, *P* = 0.02, *R*^2^ = 13.1%), but not on the skin (Pearson’s correlation, *P* = 0.88, *R*^2^ = 0.8%).

For the other parameters tested (Table S1), only milk urea nitrogen (MUN) content was significantly correlated with bacterial beta-diversity in milk (PERMANOVA, *R*^2^ = 12.96%, *P* = 0.019). MUN was significantly positively correlated with *Intrasporangiaceae* (*R*^2^ = 31.3%) and *Brevibacteriaceae* (*R*^2^ = 18.2%) and negatively correlated with *Pseudomonadaceae* (*R*^2^ = −31.3%) and *Streptococcaceae* (*R*^2^ = −11.3%) (*P* < 0.05, Pearson’s correlation). MUN was also significantly correlated with bacterial beta-diversity on the teat skin (PERMANOVA, *R*^2^ = 13.31% *P* = 0.008). MUN was significantly positively correlated with skin-associated *Brevibacteriaceae* (*R*^2^ = 24.3%) and *Intrasporangiaceae* (*R*^2^ = 19.7%) and negatively correlated with *Pseudomonadaceae* (*R*^2^ = −24.0%).

### Milk and skin microbiota differ between dairies and timepoints

Because our results strongly indicated that the microbiota detected in freshly expelled milk and on the udder teat skin were significantly different, these sites were examined separately in subsequent analyses. Comparisons within those sample types showed that the main experimental factors explaining the differences in bacterial beta-diversity were the dairy (*R*^2^ = 6.22 for milk, *R*^2^ = 7.56 for skin) and time of sampling (*R*^2^ = 4.74 for milk and *R*^2^ = 3.77 for skin) (Table 2). When examined collectively across timepoints and without taking dairy into account, CB or CH use was associated with a small but significant impact on bacterial beta-diversity in milk (Bray-Curtis, Adonis, *P* = 0.003, *R*^2^ = 1.4%). This difference with antibiotic use was not found for the skin (Bray-Curtis, Adonis, *P* > 0.05, *R*^2^= 0.8%, Table 2). Remarkably, the effect size of SCC was small and not significant (Table 2).

**TABLE 2:** Effect sizes of dairy, timepoint, treatment, and SCC on milk and skin bacterial beta-diversity

	Milk			Skin		
	*R* ^2*a*^	*F*	*P*-value^*b*^	*R* ^2^	*F*	*P*-value
Dairy	6.22	10.98	0.001	7.56	13.37	0.001
Timepoint	4.74	8.38	0.001	3.77	6.67	0.001
Treatment	1.41	1.67	0.025	0.80	0.94	0.550
SCC	0.42	1.51	0.129	0.28	0.98	0.423

^*a*^ Effect size is shown as the *R*^2^ value in %.

^*b*^ Impact of experimental factors on beta-diversity was calculated using PERMANOVA with the adonis function in the vegan package. 999 permutations were performed.

Further comparisons of the three dairies showed that the alpha-diversity (as determined by the Inverse Simpson metric) of bacteria at Dairy 3 was distinct from the other two dairies. The alpha-diversity in milk of low SCC cows was significantly higher at baseline but lower compared to the other two dairies at 55–75 DIM (Fig. S5A). For the skin, the microbiota on the teat skin of low SCC cows at Dairy 1 had the lowest alpha diversity at baseline (*P* < 0.05; Fig. S5B)*,* whereas, at the day 7 and 55–75 DIM, no significant differences between dairies were observed.

Dairy and timepoint-dependent differences in milk microbiota composition across all samples were also evident at the taxonomic level (Fig. 2; Fig. S6). Milk from Dairy 3 had the highest proportions of rare taxa (“other taxa”) at baseline and 7 days later (Fig. 3). This changed at 55–75 DIM when milk from Dairy 3 was significantly enriched in *Staphylococcaceae* (median relative abundance of 43.3% [minimum of 1.2%; maximum of 94.1%]) compared to the other two dairies (median relative abundance of 8.4% [Dairy 1] and 7.1% [Dairy 2]) (Fig. S6). Additionally, milk from Dairy 3 contained lower proportions of *Corynebacteriaceae* (median relative abundance of 1.4% [minimum of 0.04%; maximum of 14.3%]), *Lachnospiraceae* (median relative abundance of 5.3% [minimum of 0.2% and maximum of 16.2%]), and *Peptostreptococcaceae* (median relative abundance of 3.0% [minimum of 0.2%; maximum of 14.8%]) than the other two dairies at 55–75 DIM (*P* < 0.05, Wilcox test) (Fig. S6). Dairy 1 had significantly lower proportions of *Peptostreptococcaceae* at baseline (median relative abundance of 3.6% [minimum of 0.1%; maximum of 20.0%]) compared to the other two dairies (median relative abundance of 8.7% [Dairy 2] and 10.1% [Dairy 3]), and this was sustained at the 7-day time point. By comparison, *Streptococcaceae* was enriched at Dairy 1 at baseline (median relative abundance of 4.5% [minimum of 0.1%; maximum of 98.8%]) (Fig. S6).

For teat skin, there were also significant differences in the proportions of several taxa between dairies and across timepoints (Fig. 3; Fig. S7). Levels of *Corynebacteriaceae* on the cow teat skin were distinct between all three dairies at baseline, ranging from a median relative abundance of 22.3% (minimum of 1.5%; maximum of 78.8%) at Dairy 1, 3.2% (minimum of 0.8%; maximum of 8.8%) at Dairy 2, and 8.8% (minimum of 2.4%; maximum of 28.5%) at Dairy 3 (Fig. S7). The high proportions of *Corynebacteriaceae* at Dairy 1 were not sustained 7 days later, but differences between the three dairies remained. Additionally, at that timepoint, the proportions of *Peptostreptococcaceae* were significantly higher (*P* < 0.05, pairwise non-parametric Wilcoxon test) on the teat skin at Dairy 2 (median relative abundance of 9.6% [minimum of 0.6%; maximum of 21.9%]) compared to Dairy 3 (median relative abundance of 5.8% [minimum of 0.3%; maximum of 28.1%]) (Fig. S7). Finally, significant differences in *Staphylococcaceae* relative abundances were observed for teat skin at each timepoint. At baseline, teat skin swabs from Dairy 3 had a significantly higher relative abundance of *Staphylococcaceae* (median relative abundance of 9.8% [minimum of 0.7%; maximum of 29.0%]) compared to Dairy 1 (median relative abundance of 4.2% [minimum of 0.1%; maximum of 21.9%]) and Dairy 2 (median relative abundance of 3.5% [minimum of 1.2%; maximum of 77.4%]). Seven days later, Dairy 2 had the highest relative abundance of *Staphylococcaceae* (median relative abundance of 8.0% [minimum of 1.2%; maximum of 37.3%]), whereas at 55–75 DIM, both Dairy 2 and Dairy 3 had significantly higher proportions of *Staphylococcaceae* (median relative abundance of 6.8% [minimum of 0.3%; maximum of 27.5%]) than Dairy 1 (Fig. S7).

### Long-term effects of antibiotic use were minor and limited to individual dairies

Due to the small effect size of antibiotic use on the microbiota in milk (1.4%) and on the skin (0.8%) and the significant differences between dairies, the impacts of CB and CH were assessed separately for each dairy. Compared to baseline at Dairy 1 and Dairy 2, there was no significant change in bacterial alpha-diversity in milk collected from cows given CB or CH at either day 7 (CB, *P* = 0.6 and CH, *P* = 0.4, Inverse Simpson, Dunn’s test) or 55–75 DIM (CB, *P* = 0.2 and CH, *P* = 0.7). At Dairy 3, bacterial alpha-diversity (milk and teat skin) at baseline was significantly higher compared to 55–75 DIM (CB, *P* = 0.03 and CH, *P* = 0.01, for milk and CB, *P* = 0.003 and CH, *P* = 0.005, for teat skin). However, this effect could not be attributed to antibiotic treatment because the reduction in bacterial diversity was also observed for the controls (low SCC controls and high SCC controls) at that dairy.

**Fig 5:**
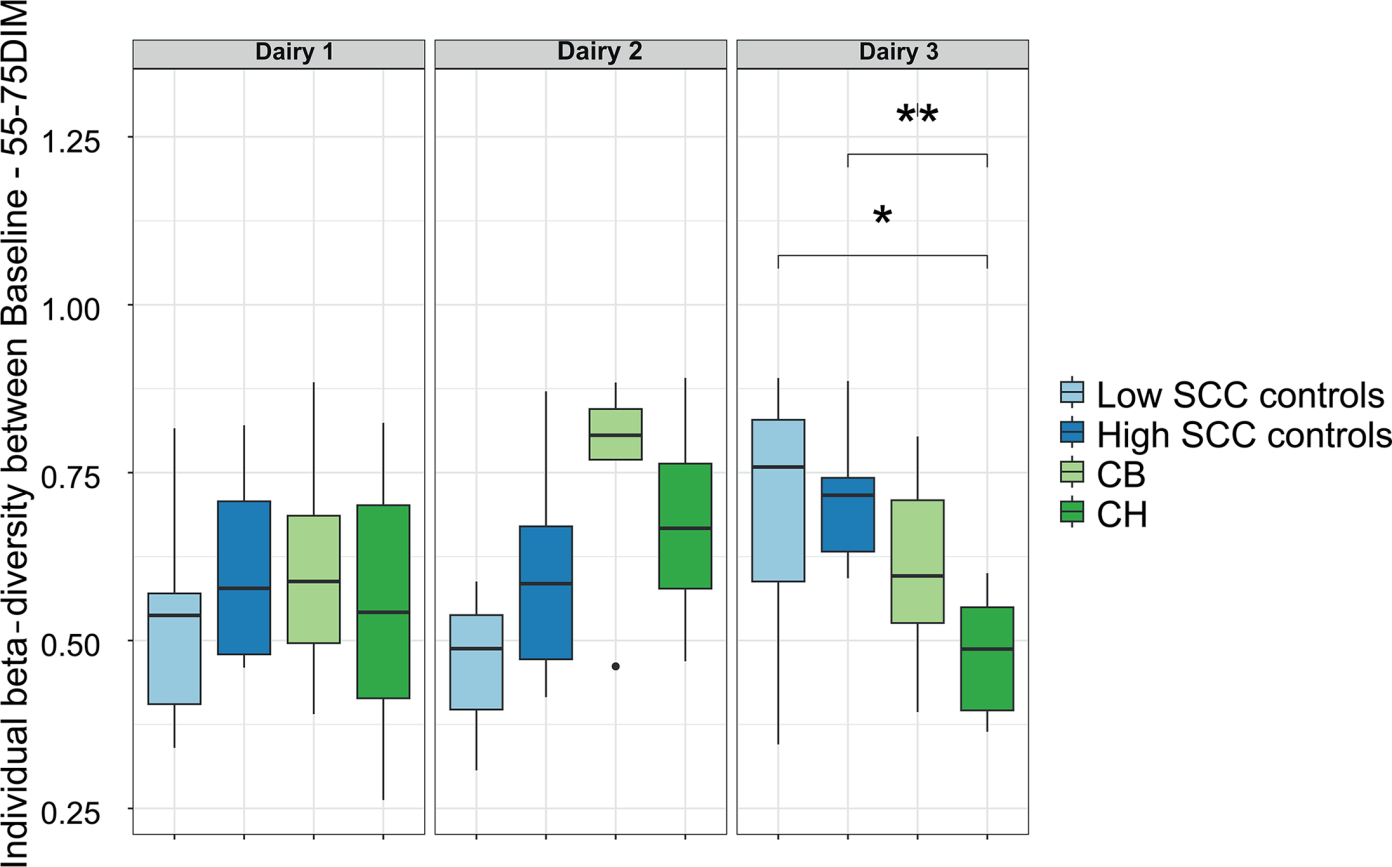
Intra-individual changes in bacterial beta-diversity between baseline and 55–75 DIM. Beta-diversity was calculated between milk collected at baseline and 55–75 DIM. A higher beta-diversity indicates a shift in microbiota composition, with 1 indicating a completely different and 0 indicating a completely identical microbiota composition. Significant differences were calculated using the pairwise Wilcoxon test with Benjamini-Hochberg multiple testing correction (*P* < 0.01 **, *P* < 0.05 *).

**Fig 6:**
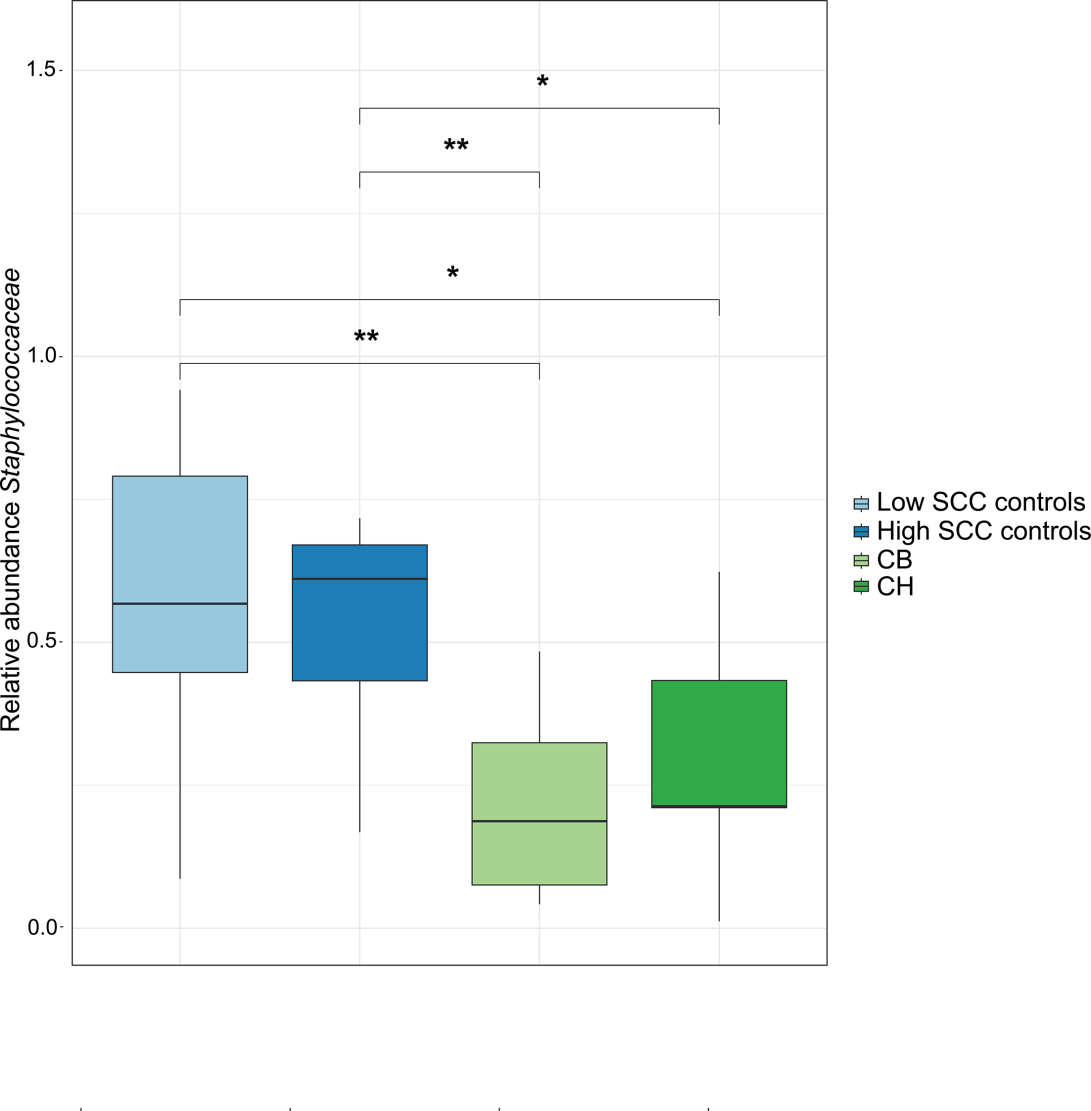
Reduced proportions of *Staphylococcaceae* in milk following antibiotic use at Dairy 3. The relative abundance of *Staphylococca*ceae in milk at Dairy 3 at 55–75 DIM is shown. Each boxplot displays the median (horizontal line) and 25% quantile and 75% quantile. Significant differences were determined with the pairwise Wilcoxon test with Benjamini-Hochberg multiple testing correction (*P* < 0.01 **, *P* < 0.05 *).

To consider the long-term effects of inter-individual variations in milk and skin, bacterial composition was compared for each cow over time (baseline and 55–75 DIM) following antibiotic use (Fig. 5). For skin, no significant differences were observed. For milk collected from Dairy 1 and 2, no significant changes in the microbiota beta-diversity were found according to this analysis, irrespective of antibiotic treatment (Fig. 5). For Dairy 3, at 55–75 DIM, milk collected from cows that received CB or CH had a smaller change in bacterial beta-diversity relative to baseline samples compared to the low and high SCC controls (Fig. 5). This could potentially be linked to the effect of antibiotics on *Staphylococcaceae* at Dairy 3, a family which was found in high levels (median relative abundance = 43.3%, min = 1.2% and max = 94.1%) in the milk at Dairy 3 at 55–75 DIM across all treatment groups. CB and CH use was associated with significant (*P* < 0.05), twofold reductions in proportions of *Staphylococcaceae* in milk at 55–75 DIM compared to untreated cows (Fig. 6).

## DISCUSSION

Understanding the effects of intramammary antibiotics on the milk and teat microbiota at dry-off is essential for developing improved husbandry practices. These practices should ultimately aim to control mammary infections while minimizing the risk of antibiotic resistance and undesirable perturbations to the mammary microbiota. In this multi-site, time course study, we found that neither of the commonly used cephalosporin antibiotics (CB or CH) resulted in extensive and repeatable changes to milk or skin microbiota on cows at dry-off that had a high SCC but were otherwise healthy without observable mastitis. Only *Staphylococcaceae* levels in milk were affected with antibiotic use according to findings from one dairy (Dairy 3) and a single time point (55–75 DIM). The results point to the importance of the dairy environment, time of sampling, and potentially the stage of milking on the diversity and proportions of bacteria present in milk and on the udder skin, and thus the need to take these factors into account when planning microbiome-related interventions. This study also suggests that using cephalosporin antibiotics at dry-off provides no significant long-term benefits nor harm to cows with high SCC and not mastitis, but may instead be useful in a more targeted approach, such as when there are unwarranted increases in *Staphylococcaceae*.

Comparisons between the milk and skin microbiota showed that they had many of the same taxa in common; however, their proportions were distinct. This finding is consistent with prior observations reporting that 70% of bacterial genera are shared between the teat skin and milk of the same cow (16). However, similarities between milk and the teat skin microbiota did not extend further. The microbiota in milk may come from various sources, including the skin and potentially other sites such as the canal and apex passage (39–41). The microbiota on the teat skin can originate from the dairy environment, bedding, among other locations (42–44). Thus, the two sites should be considered to harbor different resident bacterial populations and that the teat skin is not the only source of the bacteria detected in milk.

Otherwise, the dairy farm and the time of sampling had the greatest effect sizes on the milk and skin microbiota. Previous studies conducted in Ireland (45, 46), Italy (47), China (48), and the United States (49) indicated that environmental factors, such as geography, altitude, and seasonality, significantly influence the raw milk microbiota. In addition to geographical location, farm-specific conditions, such as housing, bedding, and management practices, also impact the milk and skin microbiota (50–53). Climate factors like weather and temperature can further influence the milk and skin microbiota at different sampling times from the same location (6, 48). For this study, the three dairies examined are located in the Central Valley of California, and so the cows experienced similar climates and feed sources, although local (dairy-specific) differences are expected.

Both milk and teat skin from the three dairies contained many taxa at a relative abundance below 1%. This observation aligns with previous reports assessing bacterial composition in raw milk (46, 54), in bulk tank milk (49), and on the cow teat skin (12, 55). However, we still found a core microbiota comprised of 11 families at all dairies that accounted for approximately 55% of the total bacteria present in each milk sample at baseline. The taxa, predominated by *Staphylococcaceae, Peptostreptococcaceae,* and *Lachnospiraceae,* were also identified as core members of the milk microbiota in other studies (46, 49, 56). A core microbiota of 14 families was also identified for the teat skin and accounted for approximately 66% of the total bacteria in those samples at baseline. The taxa, predominated by *Moraxellaceae, Corynebacteriaceae*, and *Staphylococcaceae,* were previously also described as members of the core microbiota on the cow udder (52, 57, 58). These findings show that certain bacterial families are consistent colonists of milk and teat skin irrespective of geography and environment. Interestingly, the core microbiota could be related to bovine health. Several of the same bacterial families in milk and on teat skin were significantly correlated with MUN levels. High MUN values indicate an excess of nitrogen in the feed that is not utilized and instead excreted in body fluids. A positive correlation between MUN and *Streptococcus* and other mastitis pathogens (59) indicates a reduction in normal intestinal function (59). Conversely, negative correlations were found between MUN and the proportions of *Pseudomondaceae* in milk and on skin, potentially suggesting that members of these families are associated with a healthy udder.

Contrary to our understanding of antibiotic use on the gut microbiome (60, 61), intramammary injection of CB or CH did not result in short (7 days) or long-term effects (55–75 DIM into the next lactation) on the milk or teat skin microbiota. Although there was a modest effect on bacterial beta-diversity in milk (effect size of 1.4%), this change extended to specific taxa at specific dairies. These results are consistent with a prior small-scale study on five healthy cows with low SCCs, which found no significant effects of antibiotic treatments (cephalonium dihydrate and benzathine cloxacillin) on either the alpha- or beta-diversity of the milk microbiota at dry-off, in the colostrum, or at 5 days postpartum (62). Similarly, another study with 36 non-mastitis cows housed in the same facility and administered ceftiofur hydrochloride (CH) during the dry-off period reported that there was no effect on the milk microbiota or bacterial load at 7 days postpartum, nor was there an effect on the SCC score or mastitis incidence (63). Furthermore, in cases of clinical mastitis, no difference in pathogen bacterial load was found between 40 cows given an intramammary injection of CH (64). These results collectively suggest that non-selective antibiotic therapy does not significantly alter bovine milk microbiota composition. Although we did not examine for the presence of antibiotic resistance genes, the findings from our study and others suggest that there is no lasting harmful effect of antibiotic use on the udder and milk microbiome. However, because there was also a lack of observable benefit from CB and CH use, this calls into question their prophylactic use on asymptomatic cows.

The caveat to this main outcome is that cephalosporin treatment may have impacted *Staphylococaceae* at Dairy 3, such that in the next lactation cycle (55–75 DIM), a twofold lower relative abundance of *Staphylococcaceae* was observed in the milk of cows administered the antibiotics. At that dairy, *Staphylococcaceae* were highly enriched, comprising, on average, 43.3% of the bacteria detected in milk at that time point. Because *S. aureus* and non-aureus staphylococci are common causes of bovine mastitis, the use of antibiotics may therefore have been beneficial (65). The presence of staphylococci may also indicate the development of antimicrobial resistance, as it is known that these microbes are prone to develop resistance (66, 67). However, a recent cross-country study in Europe showed a low amount of antimicrobial resistance and low presence of multi-resistance *S. aureus* present in the milk of cows with bovine mastitis (68). Thus, cows at Dairy 3 may have had a microbiome more responsive to antibiotic use, indicating that targeted antibiotic treatment of “at-risk” cows with higher proportions of specific taxa could be preferred over an untargeted approach.

Although we anticipate that our findings are robust, there are a few limitations to our study. First, we had to use different DNA extraction methods for the analysis of the milk and skin microbiota. While it was possible to obtain DNA amenable to PCR from the skin swabs using routine (column-based) extraction methods, this method was unsuccessful for raw milk despite repeated attempts, different DNA polymerases, and DNA extraction kits (data not shown). Only with incubation of milk in cetrimonium bromide (CTAB) prior to DNA extraction and subsequent use of phenol and chloroform for purification (69) was it possible to obtain amplification by PCR. CTAB is a cationic detergent that facilitates removing polysaccharides and polyphenols during DNA extraction and has been successfully employed for DNA extraction from milk (53, 70, 71) and other complex food matrices, such as olives (29). The necessity to use this more laborious approach on the freshly expressed milk was remarkable since it was not necessary in our prior studies in which raw milk was examined after cooled transport and storage (28, 72). Another technical limitation is that because of the highly diverse and low proportions of many taxa in the milk and taxa, we may have missed changes to taxa that were less abundant and variable between samples. Moreover, due to the use of the short V4 region of the 16S rRNA, our taxonomic resolution is limited. The use of long-read, full 16S rRNA amplicon sequencing or shotgun metagenomic sequencing methods, if they reduce quantities of host (bovine) DNA (73), could be a good alternative for future experiments to increase the resolution to species level. In regard to sampling and site limitations, it is possible we missed short-term (<7 days) effects of antibiotic use on the microbiota. Lastly, prior antibiotic use may have conditioned or altered the milk and teat microbiota examined here. Prophylactic antibiotic use is a common practice within California, and two of the three dairies (Dairy 1 and Dairy 2) performed prophylactic treatment with antibiotics in the previous lactation phase. Although our findings are consistent with prior studies (8, 53), it is possible that the skin and teat microbiota from cows at Dairy 1 and Dairy 2 were affected by previous antibiotic exposures.

In conclusion, milk and teat skin harbor a distinct, highly diverse microbiota that differ between dairies and time of sampling during the lactation cycle. Despite this heterogeneity, no long-term global effects of antibiotic treatment were observed. Thus, while a core microbiota is present in raw milk and on teat skin, understanding the influence of specific environmental and management factors, along with targeted antibiotic use, remains critical for supporting the health of cows at dry-off.

## Data Availability

Raw sequencing data were uploaded to the European Nucleotide Archive Sequence Read Archive (ENA-SRA) under accession number PRJEB63336.

## SUPPLEMENTAL MATERIAL

Supplemental material aem.02312-25-s0001.docx Table S1 and Fig. S1 to S7.

ASM does not own the copyrights to Supplemental Material that may be linked to, or accessed through, an article. The authors have granted ASM a non-exclusive, world-wide license to publish the Supplemental Material files. Please contact the corresponding author directly for reuse.
