# Supplementary material for: Variations in cow milk and teat skin microbiota across the lactation cycle with intramammary cephalosporin use at dry-off
Source: Appl Environ Microbiol. 2026 May 5;92(6):e02312-25. doi: 10.1128/aem.02312-25 (PMC13274448; doi:10.1128/aem.02312-25)
Supplement: Supplemental material — Table S1 and Fig. S1 to S7. [file aem.02312-25-s0001.docx]

**Supplementary Tables and Figures**

**Table S1: Associations between milk and teat skin microbiota and milk parameters.**

| **Variable** | **Variation**  **explained (R^2^)** | **P-value** |
| --- | --- | --- |
| *Milk Microbiota* |  |  |
| Solids-Non-Fat | 1.40 | 0.975 |
| **Milk Urea Nitrogen** | **12.96** | **0.019*** |
| Somatic cell counts (SCC) | 8.23 | 0.080 |
| Mitochondrial complex I | 4.85 | 0.293 |
| Mitochondrial complex V | 2.18 | 0.832 |
| Mitochondrial complex IV | 5.20 | 0.253 |
| CS | 4.04 | 0.431 |
| Lactose % | 1.57 | 0.940 |
| Protein % | 2.05 | 0.849 |
| Fat % | 1.66 | 0.939 |
|  |  |  |
| *Teat skin microbiota* |  |  |
| Solids-Non-Fat | 4.69 | 0.358 |
| **Milk Urea Nitrogen** | **13.31** | **0.008*** |
| Somatic cell counts (SCC) | 4.24 | 0.385 |
| Mitochondrial complex I | 2.02 | 0.868 |
| Mitochondrial complex V | 6.26 | 0.202 |
| Mitochondrial complex IV | 2.71 | 0.731 |
| CS | 3.18 | 0.637 |
| Lactose % | 4.62 | 0.381 |
| Protein % | 1.77 | 0.913 |
| Fat % | 7.54 | 0.087 |
|  |  |  |

Associations are based on PERMANOVA tests (adonis; vegan, 999 permutations). Significant associations are marked in bold.

**Figure S1. Overview of samples available for microbial diversity assessments.** A total of 372 milk and teat samples were collected across three dairies, three timepoints (Baseline, 7 days after treatment, and 55-75 Days in Milk in the next lactation cycle), and four treatment groups (low and high SCC controls and high SCC cows given either cephapirin (CB) or ceftiofur (CH) directly after baseline collection). The number of included cows per group is indicated in the brackets behind the name. Samples examined and included in the analysis are indicated in black (317 milk and 290 skin samples in total). The total number analyzed is provided for each group and timepoint. Samples that did not yield sufficient DNA sequence reads for further processing, due to low microbial load on the skin swabs and milk samples, are shown in light gray (37 milk and 64 skin samples in total). Cows for which sampling was incomplete for logistic- or health-related reasons (e.g. sold or died) and therefore not included in the analysis are shown in white (18 milk and 18 skin samples in total).

**
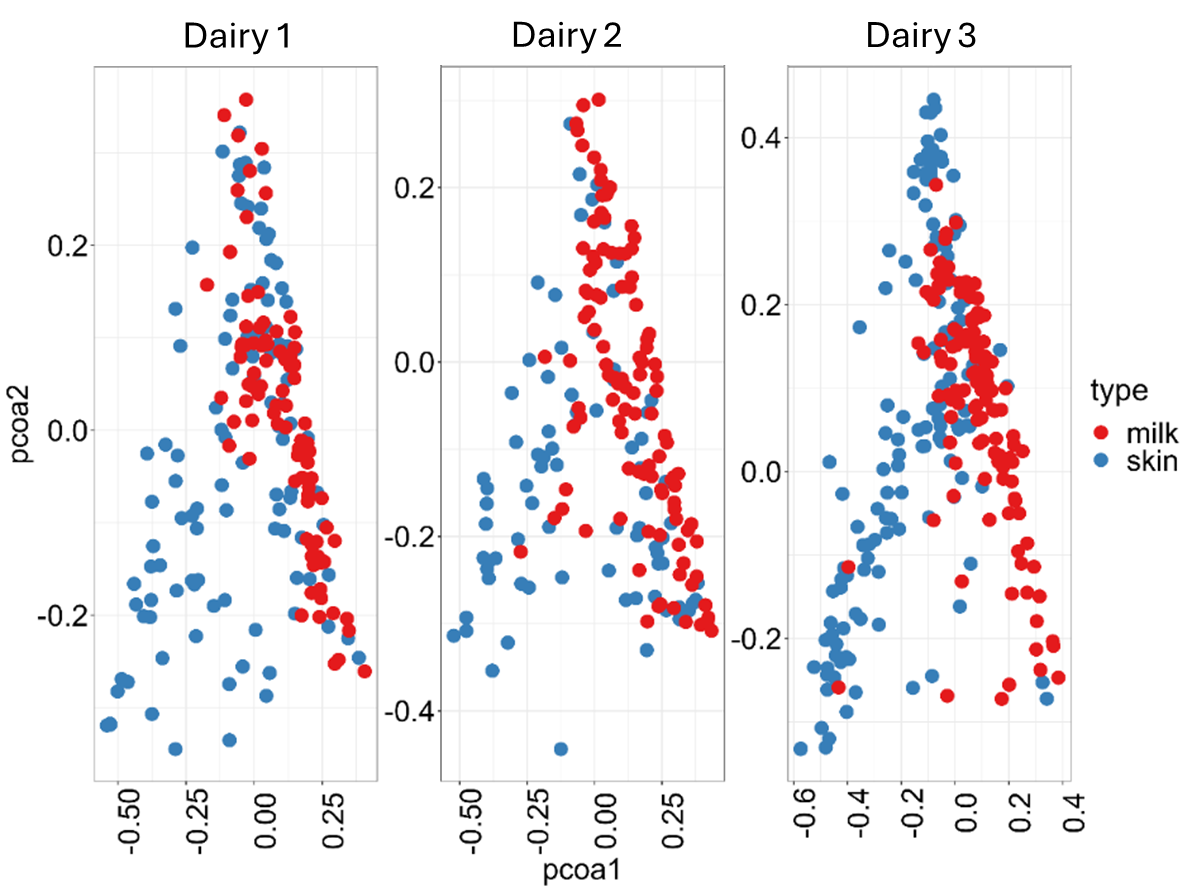
**

**Figure S2. Bacterial beta-diversity in freshly-expelled milk and on teat skin across three dairies in California.** PCoA was performed on the Bray-Curtis dissimilarity matrix. Sample type had the highest effect size (PERMANOVA < 0.05, R^2^ = 0.19).


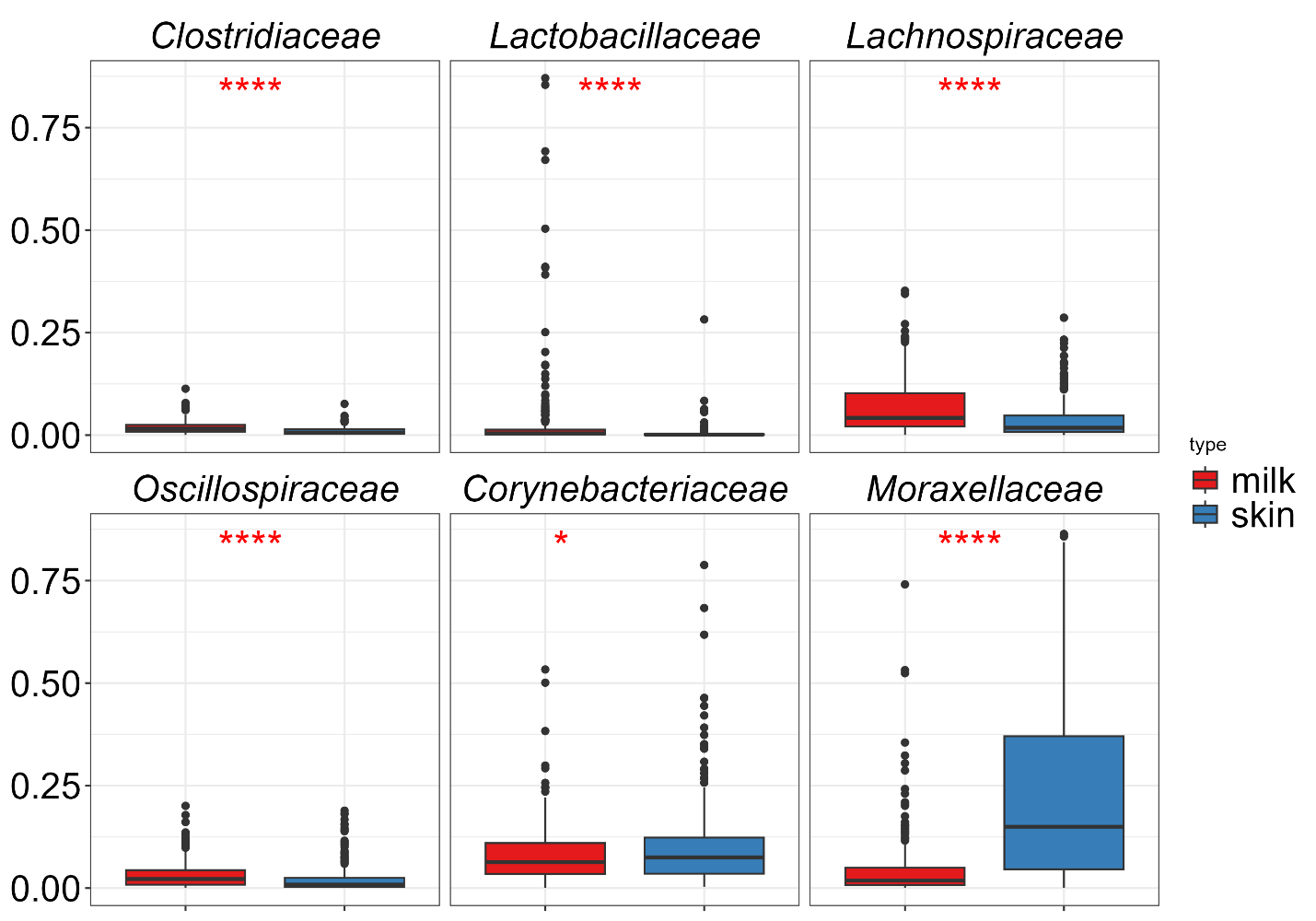


**Figure S3. Families enriched in milk or on teat skin at baseline.** *Clostridiaceae, Lactobacillaceae, Lachnospiraceae,* and *Oscillospiraceae* were enriched in milk samples collected from all dairies. *Corynebacteriaceae* and *Moraxellaceae* were enriched in skin samples collected from all dairies. Relative abundance was calculated for all cows at baseline across all dairies and significance was calculated using a Wilcoxon test (p < 0.05).

**
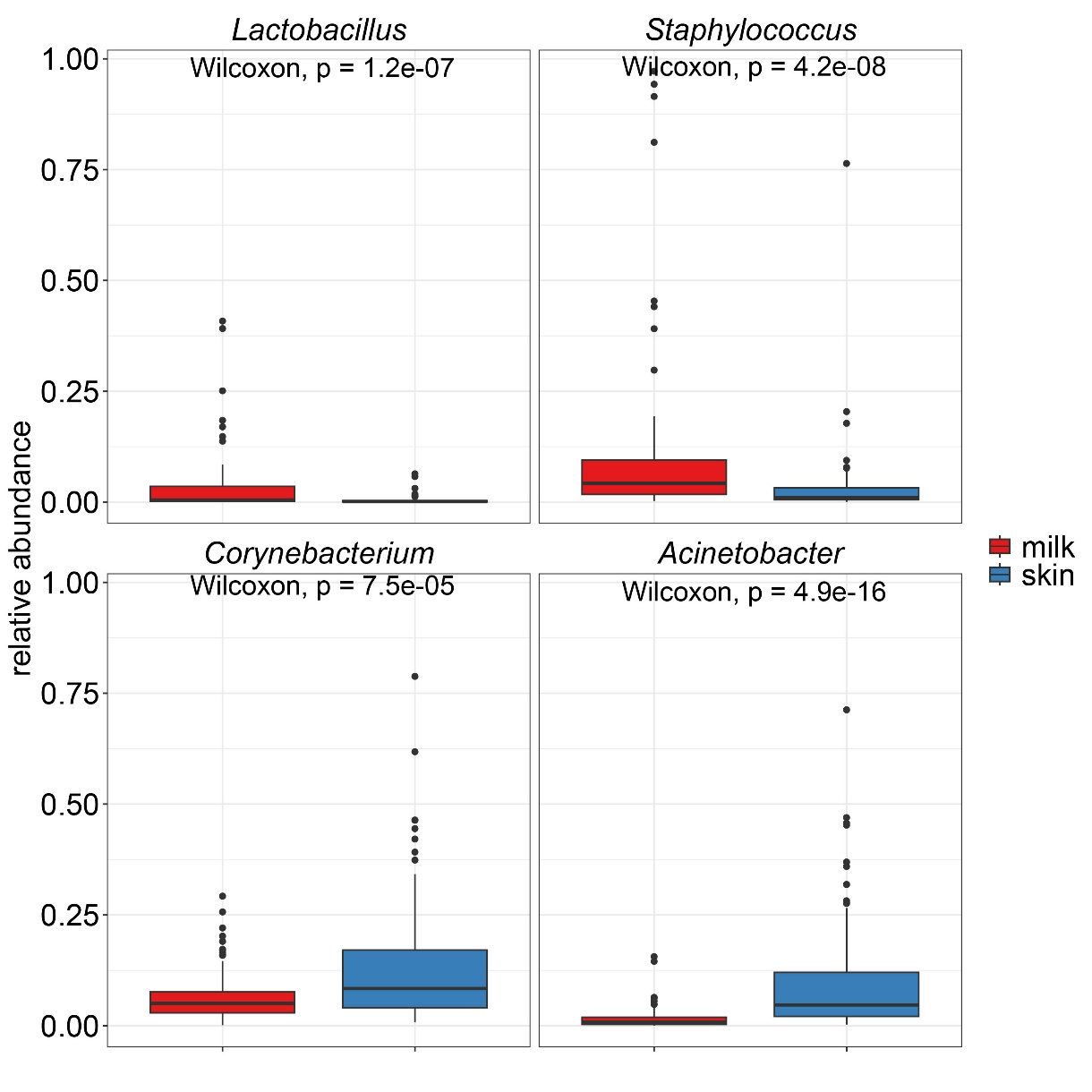
**

**Figure S4. Genera enriched in either milk or on teat skin at baseline. Shown are proportions of *Lactobacillus*, *Staphylococcus*, *Corynebacterium,* and *Acinetobacter*** in samples collected from all dairies, irrespective of SCC level. Relative abundance was calculated for all cows at baseline across all dairies and significance was calculated using a Wilcoxon test (p < 0.05).


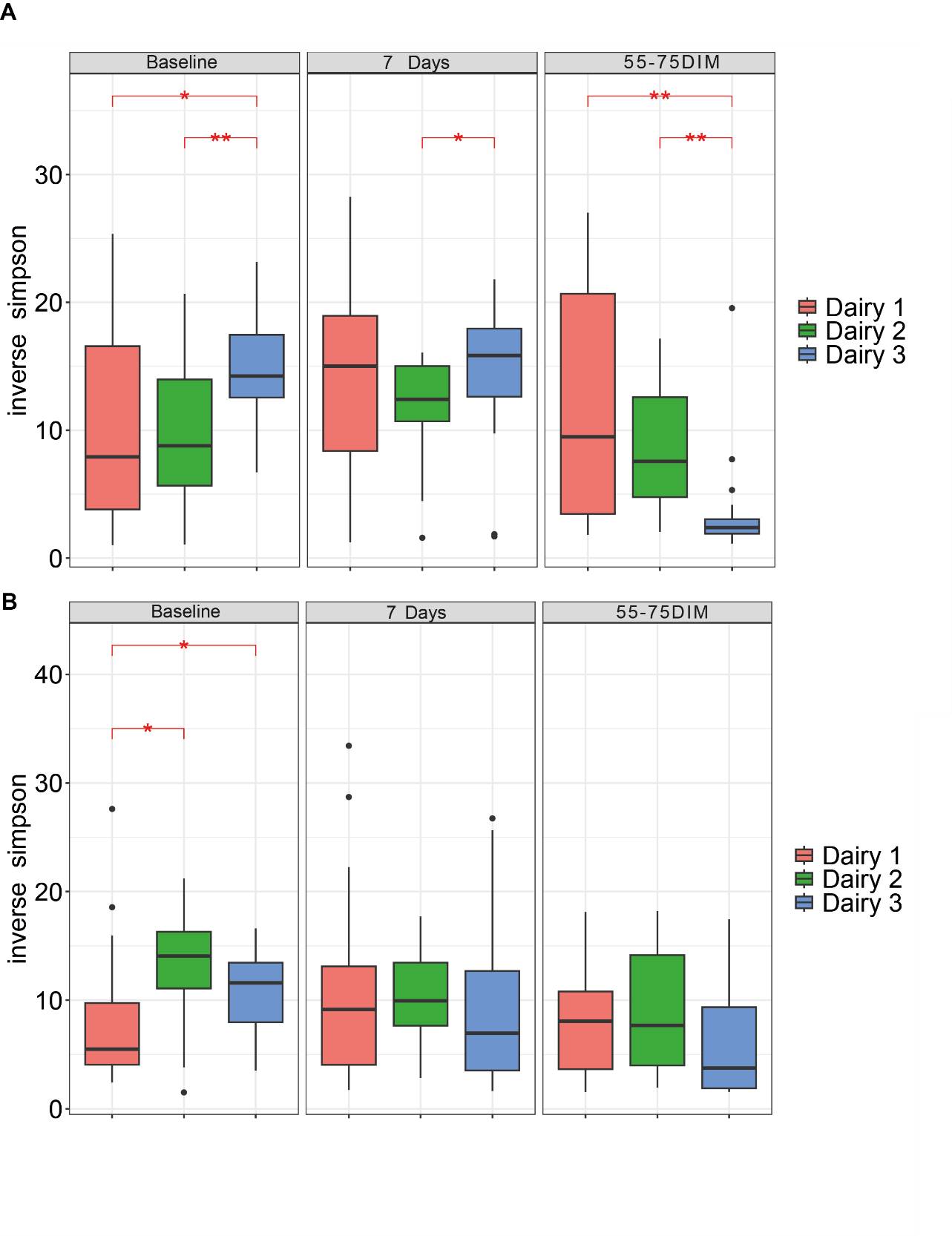


**Figure S5. Bacterial alpha-diversity compared between dairies at three timepoints.** Alpha- diversity was calculated for **(A)** milk and **(B**) skin microbiota using the inverse Simpson metric for low SCC cows at all three dairies and for each of the timepoints (baseline, 7 days and 55-75DIM). Significant differences were calculated using pairwise Wilcoxon test.

**
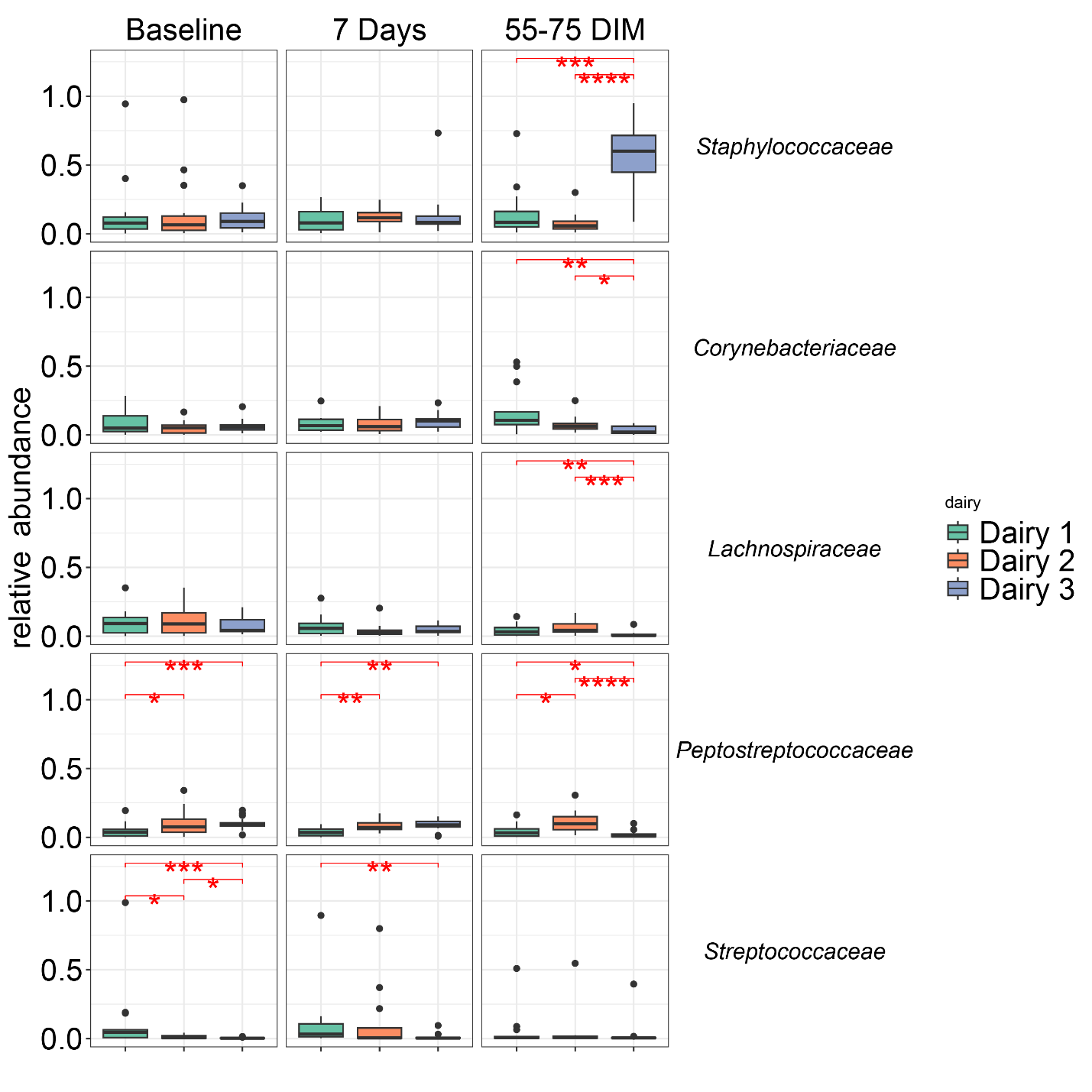
**

**Figure S6. Proportions of bacterial families in milk that differ between the three dairies.** Families detected in significantly different proportions for at least one time-point are shown. Significant differences were calculated using pairwise Wilcoxon test (* p < 0.05, ** p < 0.01, *** p < 0.001,**** p < 0.0001)


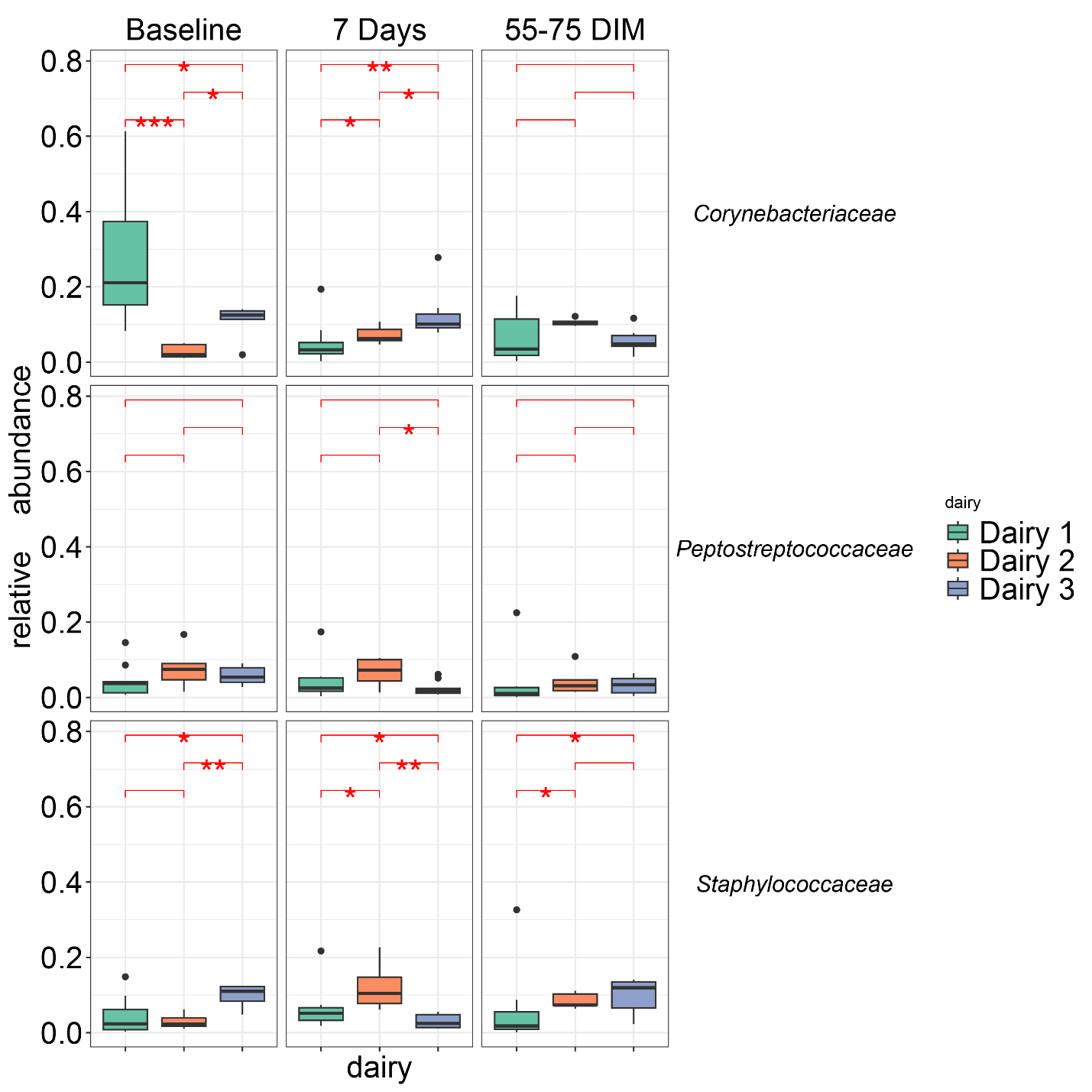


**Figure S7. Proportions of bacterial families on teat skin that differ between the three dairies.** Families detected in significantly different proportions for at least one time-point are shown. Significant differences were calculated using pairwise Wilcoxon test (* p<0.05, ** p < 0.01, *** p < 0.001,**** p < 0.0001)
